# Structural and functional analysis of the d-alanyl carrier protein ligase DltA from *Staphylococcus aureus* Mu50

**DOI:** 10.1107/S2059798322000547

**Published:** 2022-03-16

**Authors:** In-Gyun Lee, Chiman Song, Seoyeong Yang, Hanul Jeon, Jingyeong Park, Hye-Jin Yoon, Hookang Im, Sung-Min Kang, Hyun-Jong Eun, Bong-Jin Lee

**Affiliations:** a Chemical Kinomics Research Center, Korea Institute of Science and Technology (KIST), 5 Hwarangro 14-gil, Seongbuk-gu, Seoul 02792, Republic of Korea; b Department of Chemistry, Seoul National University, Seoul 08826, Republic of Korea; c Research Institute of Pharmaceutical Sciences, College of Pharmacy, Seoul National University, Seoul 08826, Republic of Korea; d College of Pharmacy, Duksung Women’s University, Seoul 01369, Republic of Korea

**Keywords:** *Staphylococcus aureus*, *dlt* operon, d-alanyl carrier protein ligase DltA

## Abstract

d-Alanylation of the teichoic acids of the Gram-positive bacterial cell wall plays crucial roles in bacterial physiology and virulence. Deprivation of d-alanine from the teichoic acids of *Staphylococcus aureus* impairs biofilm and colony formation, induces autolysis and ultimately renders methicillin-resistant *S. aureus* highly susceptible to antimicrobial agents and host defense peptides. Hence, the d-alanylation pathway has emerged as a promising antibacterial target against drug-resistant *S. aureus*. d-Alanylation of teichoic acids is mediated via the action of four proteins encoded by the *dlt* operon, DltABCD, all four of which are essential for the process. In order to develop novel antimicrobial agents against *S. aureus*, the d-alanyl carrier protein ligase DltA, which is the first protein in the d-alanylation pathway, was focused on. Here, the crystal structure of DltA from the methicillin-resistant *S. aureus* strain Mu50 is presented, which reveals the unique molecular details of the catalytic center and the role of the P-loop. Kinetic analysis shows that the enantioselectivity of *S. aureus* DltA is much higher than that of DltA from other species. In the presence of DltC, the enzymatic activity of DltA is increased by an order of magnitude, suggesting a new exploitable binding pocket. This discovery may pave the way for a new generation of treatments for drug-resistant *S. aureus*.

## Abstract 2

The crystal structure and functional characterization of DltA from *Staphylococcus aureus*, a d-alanyl carrier protein ligase that is the first protein in the d-alanylation pathway, are reported.

## 1. Introduction

*Staphylococcus aureus* is a Gram-positive bacterial human pathogen that is capable of infecting almost all human tissues and is responsible for life-threatening infections including pneumonia, endocarditis and toxic shock syndrome (Lowy, 1998 ▸; Tong *et al.*, 2015 ▸; Balasubramanian *et al.*, 2017 ▸). *S. aureus* is the leading cause of hospital-acquired infections, with substantial mortality and morbidity (Magill *et al.*, 2014 ▸; Fry & Barie, 2011 ▸). More importantly, *S. aureus* is notorious for its ability to acquire resistance to a variety of antimicrobials (Chambers & DeLeo, 2009 ▸). The emergence of methicillin-resistant *S. aureus* (MRSA) and vancomycin-resistant *S. aureus* (VRSA) has made *S. aureus*-associated infections more life-threatening and more difficult to treat. This poses a serious public health threat (Lee *et al.*, 2018 ▸; Smith *et al.*, 1999 ▸). Therefore, there is an urgent demand for novel targets and therapeutic agents to address this challenge. In an effort to develop a new antibiotic to combat multidrug-resistant *S. aureus*, we focused on the d-alanyl carrier protein ligase DltA from *S. aureus* subspecies Mu50 to provide detailed structural information that will support a rationale for novel antibiotic drug design.

The bacterial cell envelope has a unique, highly complex three-dimensional structure that plays a crucial role in maintaining cell structure and ensuring cell viability by acting as an initial barrier against external environmental stress (Seltmann & Holst, 2013 ▸). Most pathogenic bacteria, including *S. aureus*, have devised various exquisite ways of modifying their cell envelope to avoid the host defenses and to successfully infect the host. Teichoic acids (TAs) are phosphate-rich negatively charged glycopolymers that are found on the surface of a wide range of Gram-positive bacterial cell walls (Brown *et al.*, 2013 ▸; Percy & Gründling, 2014 ▸). TAs are connected to either peptidoglycan (wall teichoic acids; WTAs) or to the cytoplasmic membrane (lipoteichoic acids; LTAs) (Xia *et al.*, 2010 ▸) and play fundamental roles in bacterial physiology, resistance to antibiotics, immune evasion and pathogenesis, making TAs a promising antibacterial target (D’Elia *et al.*, 2006 ▸; Weidenmaier *et al.*, 2004 ▸; Atilano *et al.*, 2011 ▸; Kristian *et al.*, 2003 ▸; Brown *et al.*, 2012 ▸). *S. aureus* confers a net charge compensation for anionic TAs through the d-alanylation process via the action of four proteins encoded by the *dlt* operon: DltABCD (Koprivnjak *et al.*, 2006 ▸). Deprivation of d-alanine from the TAs has been shown to induce autolysis, impair biofilm formation and inhibit normal cellular growth, and ultimately renders *S. aureus* highly susceptible to antimicrobial agents (Wecke *et al.*, 1996 ▸; Koprivnjak *et al.*, 2006 ▸; Walter *et al.*, 2007 ▸; Gross *et al.*, 2001 ▸; Collins *et al.*, 2002 ▸; Peschel *et al.*, 2000 ▸; Coupri *et al.*, 2021 ▸). Thus, the d-alanylation pathway has been suggested as an attractive target for the development of novel antibiotics to treat infectious diseases caused by *S. aureus* (Weidenmaier *et al.*, 2003 ▸). d-Alanylation of TAs is a multistep process that starts in the cytoplasm, and the d-alanyl carrier protein DltA catalyzes two sequential reactions in the initial step of the process: (i) adenylation of d-alanine at the expense of ATP and (ii) thioesterification of the 4′-phosphopantetheinyl (Ppant) prosthetic group of the d-Ala carrier protein DltC (Wecke *et al.*, 1996 ▸). Through the adenylation and thioesterification reactions catalyzed by DltA, d-alanine is loaded onto the Ppant-modified conserved serine residue of DltC. d-Alanine is then transferred across the plasma membrane and incorporated into TAs through the action of two membrane-bound acyltransferases, DltB and DltD, although the detailed molecular mechanism remains elusive.

Here, we present a comprehensive structural and enzymatic characterization of the first protein in the d-alanylation pathway, DltA, from *S. aureus*. Based on comparisons of crystal structures, we detail the structural differences in the active site, as well as in the overall conformation of the enzyme, possibly explaining the discrepancies in catalytic parameters between *S. aureus* DltA and DltAs from other organisms. We believe that the characterization of DltA from the major human pathogen *S. aureus* will be valuable for future drug development and that its unique features as presented here will be exploitable when trying to rationally design specific antibiotics targeting DltA.

## 2. Materials and methods

### 2.1. Cloning, expression and purification

The cDNAs coding for *S. aureus* DltA (*Sa*DltA; UniProt P68876) and *S. aureus* DltC (*Sa*DltC; UniProt P0A018) were amplified by PCR and expressed as N-terminally His_6_-tagged fusion proteins using the expression vector pCOLD1 (Takara Bio, Shiga, Japan). The cDNA coding for *Escherichia coli* AcpS (UniProt P24224) was amplified by PCR and cloned into vector pET-28a (NEB, Ipswich, Massachusetts, USA), resulting in the production of AcpS without an affinity tag (for co-expression with wild-type *Sa*DltC), or into vector pMALc2X (for *in vitro* phosphopantetheinylation) (NEB). The S36A point mutation was introduced into *Sa*DltC using a QuikChange site-directed mutagenesis kit (Agilent Technologies, Wilmington, Delaware, USA).

*Sa*DltA and *Sa*DltC S36A were expressed in *E. coli* BL21(DE3) cells (Invitrogen, Carlsbad, California, USA). The *E. coli* cells were grown at 37°C in LB medium until the OD_600_ reached 0.5 and expression was then induced by the addition of 0.5 m*M* isopropyl β-d-1-thiogalactopyranoside. The culture was grown under inductive conditions at 15°C for 12 h. The cells were harvested by centrifugation at 8000*g*, resuspended in 50 m*M* Tris–HCl pH 8.0, 500 m*M* NaCl (buffer *A*) and lysed by ultrasonication. The resulting cell lysate was centrifuged at 20 000*g* for 1 h at 4°C. The supernatant was then bound to an Ni–NTA affinity column and eluted with buffer *A* supplemented with 300 m*M* imidazole. The His_6_ tag was removed by overnight incubation with TEV protease at 4°C and the protein was further purified using a HiLoad 16/60 Superdex 200 prep-grade column (GE Healthcare, Chicago, Illinois, USA) equilibrated with 20 m*M* Tris pH 7.5, 100 m*M* NaCl, 1 m*M* DTT. The purity of the protein was analyzed by 15% SDS–PAGE and it was concentrated to 30 mg ml^−1^ using Amicon Ultra 10 kDa molecular-weight cutoff centrifugal filters (Millipore, Burlington, Massachusetts, USA).

Wild-type (WT) *Sa*DltC was co-expressed with AcpS in *E. coli* BL21(DE3) cells to produce uniformly Ppant-modified *Sa*DltC. Recombinant plasmids containing WT *Sa*DltC and *E. coli* AcpS were co-transformed into *E. coli* BL21(DE3) and were expressed and purified similarly to as described above. The WT *Sa*DltC protein was purified using an Ni–NTA affinity column in 50 m*M* Tris pH 8.0, 500 m*M* NaCl and was eluted with 250 m*M* imidazole. The His_6_ tag was removed by overnight incubation with TEV protease at 4°C and the protein was additionally purified using a HiLoad 16/600 Superdex 75 prep-grade column (GE Healthcare) in 20 m*M* Tris pH 7.5, 100 m*M* NaCl, 1 m*M* DTT. The recombinant AcpS for *in vitro* phosphopantetheinylation was produced and purified as described previously (May *et al.*, 2005 ▸). Sequence alignments were generated with *MAFFT* (Katoh & Standley, 2013 ▸) and were illustrated using *ESPript* (Gouet *et al.*, 2003 ▸). The conservation scores of the amino acids in sequence alignments were determined with *Scorecons* (Valdar, 2002 ▸).

### 2.2. Crystallization and structure determination

Purified *Sa*DltA at a concentration of 30 mg ml^−1^ in 20 m*M* Tris pH 7.5, 100 m*M* NaCl, 1 m*M* DTT was used for initial screening of crystallization conditions. Before screening, the protein was premixed with 1 m*M* adenosine 5′-triphosphate (ATP) and 1 m*M* MgCl_2_ (Merck, Darmstadt, Germany). 1 µl protein solution was mixed with an equal volume of reservoir solution consisting of 15% PEG 4K, 0.16 *M* magnesium acetate, 0.1 *M* sodium cacodylate pH 7.5 at 4°C. The crystals of *Sa*DltA were transferred to a cryoprotectant solution containing 20%(*v*/*v*) glycerol for a few seconds before being cooled in liquid nitrogen. A set of X-ray diffraction data was collected at 100 K using an ADSC Quantum 315r CCD detector system (Area Detector Systems Corporation, Poway, California, USA) at the BL-7A experimental station of Pohang Light Source, Republic of Korea. The *Sa*DltA crystal belonged to the monoclinic space group *P*2_1_, with unit-cell parameters *a* = 89.46, *b* = 88.51, *c* = 130.85 Å, α = γ = 90.00, β = 91.67°. The raw data were processed and scaled using *HKL*-2000 (Otwinowski & Minor, 1997 ▸). Table 1 ▸ summarizes the data-collection statistics.

### 2.3. Pyrophosphate-detection assay

The adenylation activity of recombinant *Sa*DltA was determined using a pyrophosphate-detection assay. All of the reagents used in this assay were purchased from Merck. Assays were performed with 5 µ*M* *Sa*DltA, 5 units ml^−1^ in­organic pyrophosphatase, 5 m*M* ATP, 100 m*M* KCl, 10 m*M* MgCl_2_, 50 m*M* Tris–HCl buffer pH 7.4 and various concentrations of alanine at 37°C. The enzyme reaction was initiated by the addition of alanine, and 20 µl reaction solution was retrieved every 2 min. The retrieved reaction solution was immediately mixed with 380 µl dye solution consisting of 0.033%(*w*/*v*) malachite green and 1.3%(*w*/*v*) ammonium molybdate in 1.0 *M* HCl and incubated for 90 s. The absorbance at 620 nm was measured using a BioSpectrometer (Eppendorf, Hamburg, Germany) and the absorbance without 5 µ*M* *Sa*DltA was used as a blank. Determination of the adenylation activity of *Sa*DltA in the presence of WT or S36A mutant *Sa*DltC was performed essentially as described above. Specifically, the reaction was conducted in the presence of *Sa*DltA (5 µ*M*), 5 m*M* d-alanine and 300 µ*M* WT or S36A mutant *Sa*DltC. The initial rates of the enzyme reaction were derived from the time courses of phosphate accumulation. Rate–concentration curves against alanine were fitted to the Michaelis–Menten equation using *Prism* 6 (GraphPad, San Diego, California, USA) to obtain kinetic parameters.

### 2.4. Data deposition

Atomic coordinates and structure-factor amplitudes for the structure of *Sa*DltA have been deposited in the Protein Data Bank (PDB) with accession code 7vhv.

## 3. Results

### 3.1. Overall structure of *S. aureus* DltA

**Figure 1:**
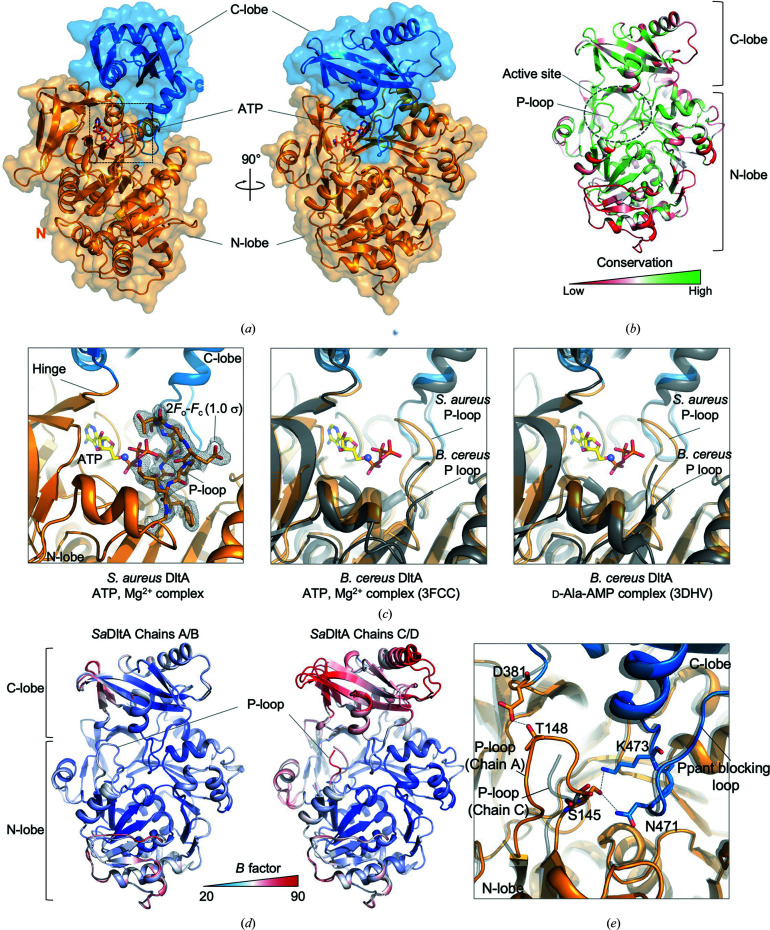
Overall structure of *Sa*DltA. (*a*) Ribbon diagram and surface representation of two perpendicular views of *Sa*DltA (the N-terminal lobes and C-terminal lobes are colored orange and blue, respectively). The ATP molecule bound in the active site is shown using a stick representation and is colored by atom type. (*b*) Sequence conservation of *Sa*DltA (see also Fig. 2 ▸) mapped onto the surface of the structure and colored from low to high conservation using a red to green gradient. (*c*) Left: close-up view showing the ATP- and Mg^2+^-bound active site of *Sa*DltA. The 2*F*_o_ − *F*_c_ electron-density map (gray mesh) contoured at 1.0σ is shown around an all-atom representation of the P-loop. Center: close-up view showing the ATP- and Mg^2+^-bound active site of *B. cereus* DltA (PDB entry 3fcc). Right: close-up view showing the d-alanine-adenylate-bound active site of *B. cereus* DltA (PDB entry 3dhv). (*d*) *B*-­factor distribution mapped onto the surface of *Sa*DltA chains *A* and *B* (left) or chains *C* and *D* (right). The *B*-factor distribution is colored from low (20 Å^2^) to high (90 Å^2^) using a blue to red gradient. (*e*) Close-up view showing the interaction between the P-loop and residues residing in the hinge region of the Ppant blocking loop. Chain *A* of *Sa*DltA is colored orange (N-lobe) and blue (C-lobe) and chain *C* of *Sa*DltA is colored gray.

DltA from *S. aureus* (*Sa*DltA) was crystallized in the presence of ATP and Mg^2+^. The structure was solved by the molecular-replacement method employing ATP-complexed *B. cereus* DltA (PDB entry 3fcc; Osman *et al.*, 2009 ▸) as the search model (Table 1 ▸). The monoclinic crystal, belonging to space group *P*2_1_, contains four DltA molecules (chains *A*, *B*, *C* and *D*) in the asymmetric unit. Two of the complexes (chains *C* and *D*) are relatively poorly defined in the electron-density map, yet fourfold noncrystallographic symmetry (NCS) averaging was used throughout the initial steps of the refinement, yielding well defined electron density for each crystallographic unit. Analysis of the protein interfaces in the asymmetric unit did not show specific interactions that would indicate the formation of oligomers; the complexation significance score (CSS), which indicates the significance of the interface for assembly formation, was 0.000. The atomic model of *Sa*DltA has been refined to a crystallographic *R* factor of 0.189 (*R*_free_ = 0.239; Table 1 ▸) and features a characteristic ‘adenylation conformation’ (Fig. 1 ▸ *a*).

Similar to other adenylate-forming enzymes (Schmelz & Naismith, 2009 ▸), DltA utilizes several distinct structural conformations to catalyze two sequential reactions: (i) adenylation of d-Ala at the expense of ATP followed by (ii) thioesterification of the 4′-phosphopantetheinyl (Ppant) prosthetic group of the d-Ala carrier protein DltC (Supplementary Fig. S1*a* ). Upon substrate binding by the substrate-free ‘open conformation’ (Gulick, 2009 ▸; Weissman, 2015 ▸), binding of ATP and the d-Ala substrate facilitates the structural change of the enzyme towards the ‘adenylation conformation’ by inducing electrostatic changes in the active-site pocket and mutual approach of the N-terminal and the C-terminal lobes, closing the active site from the bulk solvent and completing the active site capable of d-Ala adenylation. Subsequent d-Ala adenylate formation and breakage of the ATP phosphodiester bond result in a 140° rotation of the C-terminal subdomain towards the ‘thiolation conformation’, in which the Ppant group of DltC can approach the active site of DltA for loading of d-Ala to complete the reaction (Du *et al.*, 2008 ▸; Osman *et al.*, 2009 ▸; Du & Luo, 2014 ▸). The *Sa*DltA structure features the canonical adenylation conformation, allowing it to grasp the ATP substrate and Mg^2+^ ion in the active site that lies between the N- and C-terminal lobes and posing it for the d-alanine adenylation reaction with the Ppant substrate channel blocked by the loop (Ppant blocking loop; residues 463–476) in the C-terminal lobe (Figs. 1 ▸ *a* and 1 ▸ *c*).

*Sa*DltA consists of two structurally distinct lobes: an N-terminal lobe (residues 1–377), a C-terminal lobe (residues 382–485) and a flexible interdomain hinge region (residues 378–381) connecting the two lobes (Figs. 1 ▸ *a* and 2 ▸). The flexible nature of the hinge region, consisting of four highly conserved residues (Gly-Arg-Ile-Asp), allows the proper relative orientation of the N- and C- lobes for the adenylation conformation and thiolation conformation (Yonus *et al.*, 2008 ▸). Clear electron density for ATP and magnesium in the active-site pocket is observed in all chains in the asymmetric unit (Supplementary Fig. S1*b* ). The active-site pocket is positioned in a deep cleft between the N- and C- terminal lobes of DltA formed by conserved amino-acid residues mostly from the large N-terminal domain, including the highly conserved P-loop (Figs. 1 ▸ *a*, 1 ▸ *b* and 2 ▸). Interestingly, we first observed a clear density for the P-loop of DltA in the adenylation conformation, which has not been observed in other DltA structures (Figs. 1 ▸ *c* and 1 ▸ *d*), as discussed in detail below.

The characteristic structural features between chains were identified when we superimposed the four *Sa*DltA monomers in the asymmetric unit (Fig. 1 ▸ *e* and Supplementary Fig. S2). The most prominent difference between chains is the structural stability of the aforementioned P-loop (*S. aureus* residues ^144^Thr-Ser-Gly-Ser-Thr-Gly-Glu-Pro-Lys^152^) and interactions involving the P-loop that covers the one side of the active site. The P-loop of DltA has an amino-acid composition similar to that of the P-loop or Walker A motif found in other adenylate-forming enzymes (Schmelz & Naismith, 2009 ▸); thus, the loop has been suggested to bind ATP or pyrophosphate, despite the lack of direct structural data to support this assumption. In chains *A* and *B* of *Sa*DltA we observed clear electron density allowing us to unambiguously assign the residues of the P-loop (Fig. 1 ▸ *c*), whereas the electron density of the corresponding residues in chains *C* and *D* was very weak and we could not model four residues (residues 148–151), indicating significant flexibility of the region (Fig. 1 ▸ *d*). In chains *A* and *B* the P-loop directly interacts with the hinge-region residue Asp381, as well as the C-terminal domain residues Asn471 and Lys473 that reside in the Ppant blocking loop, while the corresponding interactions are absent in chains *C* and *D* (Figs. 1 ▸ *d* and 1 ▸ *e*). Conceivably, the observed interactions involving P-loop residues are likely to stabilize the overall conformation of the protein by generating a more compressed and stable structure in which the C-terminal lobe moves towards the N-terminal lobe (Supplementary Fig. S2). Indeed, the C-terminal lobes of chains *A* and *B* have nearly identical structures [root-mean-square (r.m.s.) deviations of 0.38 Å between chains *A* and *B*] with an average *B* factor of 45–50 Å^2^, whereas the C-terminal lobes of chains *C* and *D*, in which the P-loops are disordered, reveal a variety of conformations (r.m.s. deviations of 1.56 Å between chains *C* and *D*) with an average *B* factor of 64–73 Å^2^ (Fig. 1 ▸ *d*). Interestingly, the previously reported substrate-free structure of *B. cereus* DltA (Du & Luo, 2014 ▸) shows a noticeably more flexible structure than the other adenylation-conformation structure of DltA, and the disordered region observed in the apo structure (*B. cereus* residues Arg397–Glu413 and Lys433–Tyr440) coincides well with the region that shows structural variation in *Sa*DltA chains *C* and *D* (*S. aureus* DltA β22–β23 and β24–β25), in which the P-loops are disordered (Supplementary Fig. S2). These observations indicate that the binding of the P-loop to the β- and γ-phosphates of the ATP molecule, as well as binding to the Ppant blocking loop and hinge region, may be a prerequisite for the adenylation reaction by promoting the structural transition from the apo state to the adenylation state (Supplementary Fig. S2*b* ; Du & Luo, 2014 ▸; Chen *et al.*, 2015 ▸).

### 3.2. *Sa*DltA shows distinct structural features compared with other DltA structures

There are several previously published DltA structures in the literature from *B. cereus* and *B. subtilis*. The structures from *B. cereus* are in the adenylation conformation [PDB entries 3fcc (complexed with ATP and Mg^2+^), 3fce (complexed with ATP and Ca^2+^), 3dhv (complexed with d-Ala-adenylate) and 4pzp (apo)] (Du & Luo, 2014 ▸; Osman *et al.*, 2009 ▸), while a structure from *B. subtilis* features the thiolation conformation (PDB entry 3e7w; complexed with AMP; Yonus *et al.*, 2008 ▸).

While the overall architecture of *Sa*DltA is similar to those of previously reported *B. cereus* DltA structures in the adenylation conformation, several discrete structural dissimilarities were identified in *Sa*DltA, especially in the interface between the large and small lobes, in addition to the aforementioned distinct structural features of the P-loop.

**Figure 2:**
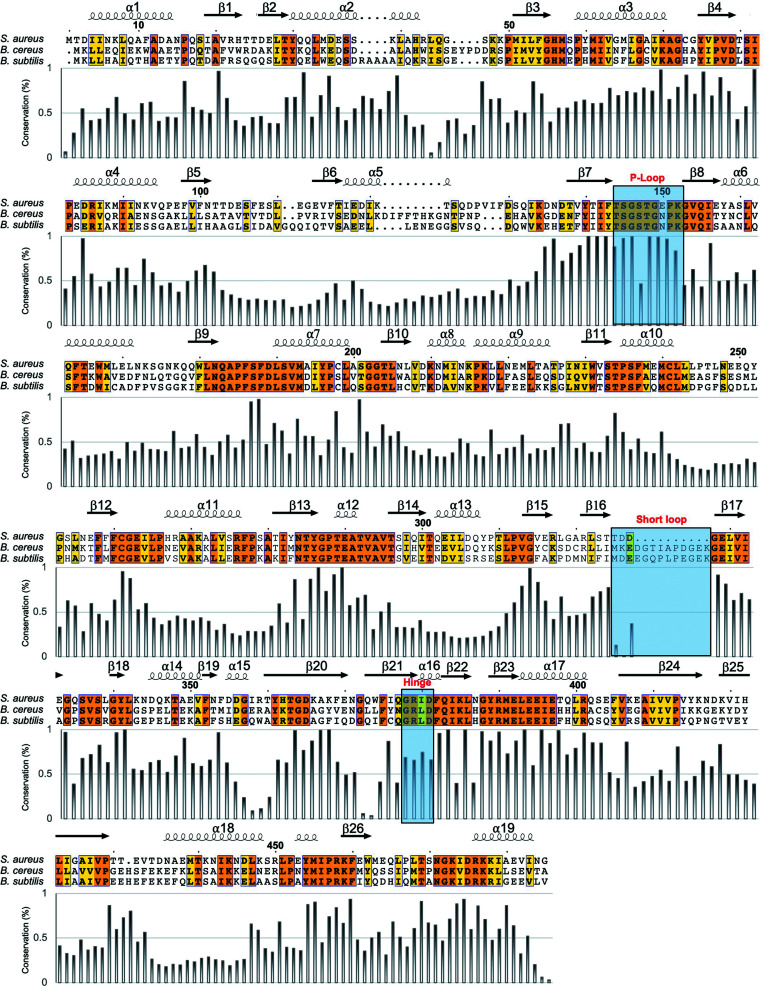
Alignment of DltA from *S. aureus*, *B. cereus* and *B. subtilis*. Orange backgrounds indicate 100% sequence identity and yellow backgrounds indicate amino acids with similar physicochemical properties. The P-loop, hinge region and unique short loop of *S. aureus* discussed in the text are marked with blue boxes. Sequence-conservation scores were calculated from the alignment of 100 DltA sequences from different species using *Scorecons* (Valdar, 2002 ▸). The resulting per-residue scores were then plotted against the *S. aureus* DltA sequence.

**Figure 3:**
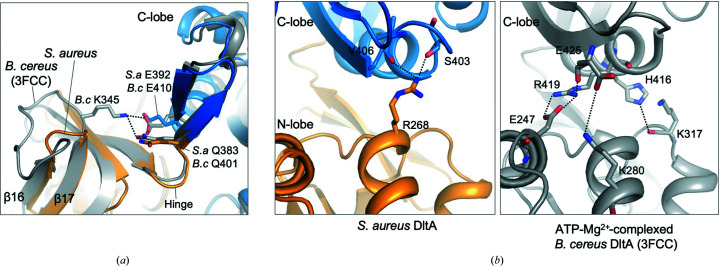
Several key interdomain interactions are absent in *Sa*DltA. (*a*) Close-up view of the short loop (Thr325–Gly328) linking β-strands β16 and β17 of *Sa*DltA (orange and blue, chain *A*) and the equivalent region in *B. cereus* (gray). Hydrogen-bond interactions are shown as dotted lines. Note that the interaction of Lys345 with Glu410 and Gln401 is absent in the structure of *S. aureus* DltA. (*b*) Left: close-up view of the hydrogen-bond interactions between Arg268 in the N-lobe and Ser403 and Val405 in the C-lobe of *Sa*DltA (orange and blue, chain *A*). Right: close-up view of the hydrogen-bond interactions observed in the equivalent region of *B. cereus* DltA (gray; PDB entry 3fcc). Hydrogen-bond interactions are shown as dotted lines.

Interestingly, in *Sa*DltA we observed many fewer inter­actions between the large N-lobe and the small C-lobe compared with DltA from *B. cereus* (Fig. 3 ▸). In *Sa*DltA a very short loop (Thr325–Gly328) links the two β-strands β16 and β17, in comparison to 12 residues that loop out in other DltAs (Figs. 2 ▸ and 3 ▸ *a*). The observed differences in the length of the loop region lead to substantial differences in the interdomain interactions formed between this structural element and the C-terminal lobe of the protein. In *B. cereus* DltA the positively charged Lys345 positioned in the loop makes hydrogen-bond interactions with the highly conserved Glu401 and Glu410 in an antiparallel hairpin right next to the hinge residues. However, these interactions are missing in *Sa*DltA (Fig. 3 ▸ *a*). Although the linker is inherently flexible, a degree of rigidity is introduced and may serve to keep the antiparallel hairpin region tethered, therefore stabilizing the overall adenylation conformation of the protein.

In addition, several other key hydrophilic interdomain interactions are missing in *Sa*DltA. In structures of *B. cereus* DltA in the adenylation conformation, interactions between the N- and C-lobes are predominantly mediated by hydrogen bonds/salt bridges. In *B. cereus* DltA, the Arg419, Glu425 and His416 (*B. cereus* numbering) residues in the C-terminal lobe highly stabilize the interface between the N- and C-lobes by forming salt bridges/hydrophilic interactions with Glu247, Lys280 and Lys317 on the N-terminal lobe, respectively (Fig. 3 ▸ *b*). However, these interactions are absent in *S. aureus* DltA, and the only interactions observed in the region are the hydrogen bonds between the backbone of Ser403/Val406 and the side chain of Arg268 (Fig. 3 ▸ *b*). The absence of this interaction is likely to affect the adenylation–substrate-free conformational equilibrium of DltA and could reduce the catalytic efficiency of the enzyme, as the DltA–substrate interactions may be used to compensate for the energetic cost of stabilizing the adenylation state of the enzyme. Indeed, the catalytic efficiency of the DltA enzyme for the adenylation reaction is lower than that of previously reported DltAs (as discussed in detail below), but the substrate specificity is higher.

### 3.3. ATP-binding pocket of *Sa*DltA

**Figure 4:**
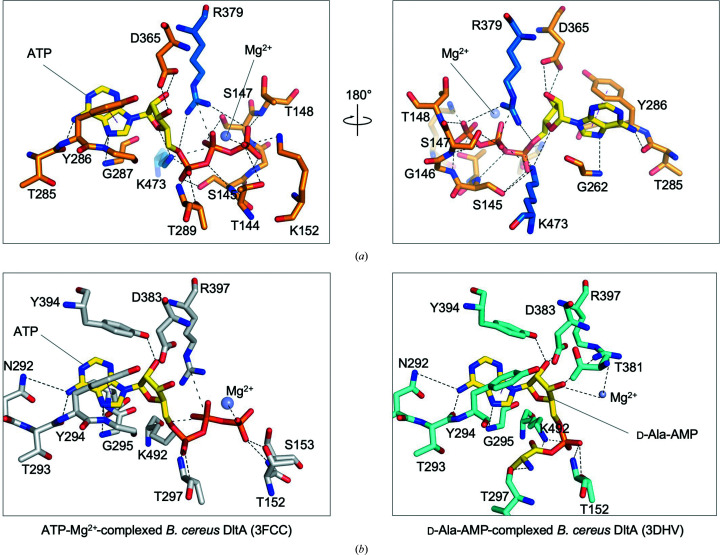
Detailed active-site structures of *S. aureus* DltA and *B. cereus* DltA. (*a*) Close-up view of the active site of *S. aureus* DltA (chain *A*). The bound Mg^2+^ ion is shown as a purple sphere. The ATP molecule and the key residues engaged in the hydrogen-bonding network are shown as stick models. Residues from the N- and C-lobes are colored orange and blue, respectively. (*b*) Left: close-up view of the active site of ATP- and Mg^2+^-complexed *B. cereus* DltA (PDB entry 3fcc). Residues engaged in the hydrogen-bonding network are shown as stick models and colored white. Right: close-up view of the active site of d-­alanine-adenylate-complexed *B. cereus* DltA (PDB entry 3dhv). Residues engaged in the hydrogen-bonding network are shown as stick models and colored cyan.

The active-site pocket of *Sa*DltA is positioned in a deep cleft between the N- and C-terminal lobes formed by conserved amino-acid residues mostly from the large N-terminal domain, including the highly conserved P-loop (Fig. 4 ▸ *a*). As mentioned earlier, we observed electron density for the P-loop in chains *A* and *B* of *Sa*DltA and we are limiting our discussion to chain *A* of the molecule in the asymmetric unit, as chain *A* shows the clearest density map. The overall architecture of the *Sa*DltA active site resembles that of DltA from *B. cereus* in the adenylation state, and characteristic key conserved residues essential for substrate binding and the adenylation reaction are present in the active site of *Sa*DltA (Figs. 4 ▸ *a* and 4 ▸ *b*). The planar adenosine ring of ATP forms extensive hydrophilic interactions with highly conserved residues including Gly287, Tyr286, Thr285 and Gly262 (Fig. 4 ▸ *a*). In *Sa*DltA the adenosine ring makes π-stacking interactions with the aromatic side chain of Tyr286. The ribose moiety of ATP is held in place by hydrogen bonds to the side chains of Asp365 and Lys473. While the residues involved in the interaction between the adenosine ring and ribose parts of the bound ATP in *Sa*DltA are similar to those of *B. cereus* DltA in the adenylation conformation (Fig. 4 ▸ *b*), we observed unique features around the β- and γ-phosphate parts of the bound ATP. In *Sa*DltA the P-loop folds over the phosphate parts of the nucleotide and wraps around the phosphate moiety to form an extensive hydrogen-bonding network, presumably positioning the phosphates of ATP for catalysis (Fig. 4 ▸ *a*). The hydrogen-bonding network between the loop and phosphate includes hydrophilic interaction clusters consisting of Mg^2+^, Thr144, Ser145, Gly146, Ser147, Thr148 and Lys152. The hydrogen-bonding network between the β- and γ-phosphate parts of the bound ATP and the P-loop may help to maintain structural rigidity and stabilize ionization states to modulate the turnover rate and control the local charge balance during the adenylation reactions.

The d-alanine-binding pocket of *Sa*DltA closely resembles that of DltA from *B. cereus*. In the d-Ala-AMP-bound *B. cereus* DltA structure (Du *et al.*, 2008 ▸), the d-alanyl amino group makes hydrogen bonds to Thr297, Asp197 and Val301 and the d-alanyl methyl group points towards the small hydrophobic cavity formed by the side chains of Leu197, Met200 and Cys268. In particular, Cys268 has been shown to play a critical role in enantiomer selection, as its thiol group causes a steric clash with l-alanine but not with d-alanine (Du *et al.*, 2008 ▸; Yonus *et al.*, 2008 ▸). The corresponding residues involved in d-alanine binding are highly structurally conserved in *Sa*DltA (Supplementary Fig. S3), suggesting that *Sa*DltA would also prefer d-alanine as a substrate, as experimentally shown in the next section.

### 3.4. *S. aureus* DltA prefers d-alanine over l-alanine

**Figure 5:**
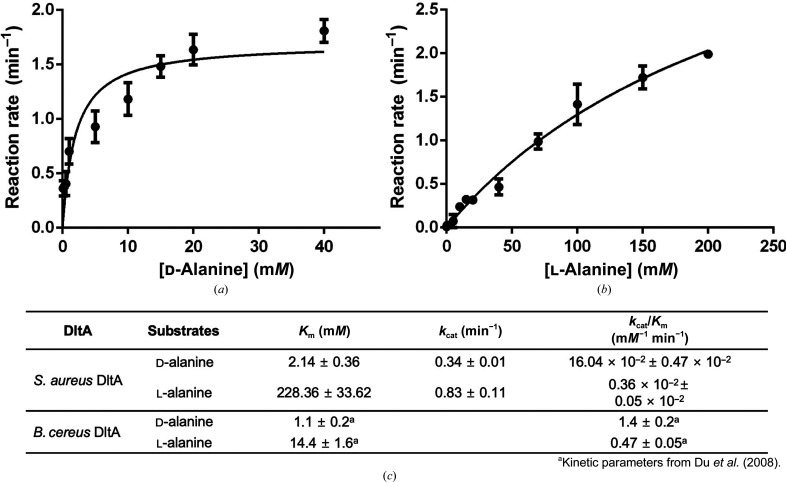
Rate–concentration curves of the adenylation catalyzed by *S. aureus* DltA in the presence of d- or l-alanine. (*a*) Adenylation reaction with various concentrations of d-alanine. (*b*) Adenylation reaction with various concentrations of l-alanine. (*c*) Kinetic parameters for *Sa*DltA. The average values of triplicate measurements and standard deviations are shown.

*B. cereus* DltA prefers d-alanine over l-alanine as a substrate (Du *et al.*, 2008 ▸). To investigate whether *Sa*DltA also prefers d-alanine, we measured the initial rates of the adenylation reaction catalyzed by *Sa*DltA via pyrophosphate-detection assays. The initial rates were measured in the presence of various concentrations of d- or l-alanine, and the rate–concentration curves towards alanine were fitted to the Michaelis–Menten equation (Figs. 5*a* ▸ and 5 ▸ *b*). The calculated kinetic parameters are listed in Fig. 5 ▸(*c*). The *K*_m_ of *S. aureus* DltA against d-alanine was lower than the *K*_m_ against l-alanine, and the catalytic efficiency (*k*_cat_/*K*_m_) against d-alanine was higher than the *k*_cat_/*K*_m_ against l-alanine. These results show that *Sa*DltA prefers d-alanine over l-alanine. For *Sa*DltA, the change in *K*_m_ (for d-alanine over l-alanine) was approximately 106.71-fold, while according to previous studies it was about 13.1-fold for *B. cereus* DltA (Du *et al.*, 2008 ▸). This indicates that the substrate specificity of *Sa*DltA is much higher than that of *B. cereus* DltA. Although the catalytic efficiency (*k*_cat_/*K*_m_) of *B. cereus* DltA was higher than that of *Sa*DltA, the change in *k*_cat_/*K*_m_ (for d-alanine over l-alanine) for *Sa*DltA was higher than that of *B. cereus* DltA.

### 3.5. The Ppant group of *S. aureus* DltC is necessary for the activation of *S. aureus* DltA

The Ppant group of DltC is required for the second reaction step catalyzed by DltA (Heaton & Neuhaus, 1994 ▸), and its mimic coenzyme A has been shown to increase the adenylation activity of *B. cereus* DltA (Osman *et al.*, 2009 ▸; Du & Luo, 2014 ▸). In order to examine whether *S. aureus* DltC (*Sa*DltC) increases the adenylation activity of *Sa*DltA and whether the Ppant group of DltC is necessary for activation, we measured the initial rates of the adenylation reaction catalyzed by *Sa*DltA in the presence of wild-type (WT) *Sa*DltC and the S36A DltC mutant. The S36A DltC mutant is incapable of being post-translationally modified with a Ppant group at the conserved Ser36 residue (May *et al.*, 2005 ▸). To produce uniformly Ppant-group-modified WT *Sa*DltC, we co-expressed WT DltC with acyl carrier protein synthase (AcpS), which catalyses the Ppant-modification reaction at Ser36 of *Sa*DltC. To confirm that the residue responsible for the Ppant modification is Ser36, we performed an *in vitro* phosphopantetheinylation reaction using purified AcpS and WT *Sa*DltC or S36A *Sa*DltC, and analyzed it using native PAGE. WT *Sa*DltC purified from *E. coli* migrated as two bands on conformationally sensitive native PAGE, as reported previously (Wood *et al.*, 2018 ▸), and shifted to a single band upon AcpS-catalyzed phosphopantetheinylation (Supplementary Fig. S4*a* ). In contrast, S36A *Sa*DltC purified from *E. coli* migrated as a single band and did not show a band shift upon AcpS-catalyzed phosphopantetheinylation, confirming that the residue responsible for the Ppant modification of *Sa*DltC is Ser36. The uniformity of modification was verified by matrix-assisted laser desorption/ionization (MALDI) time-of-flight (TOF) mass spectrometry (Supplementary Fig. S4*b* ).

**Figure 6:**
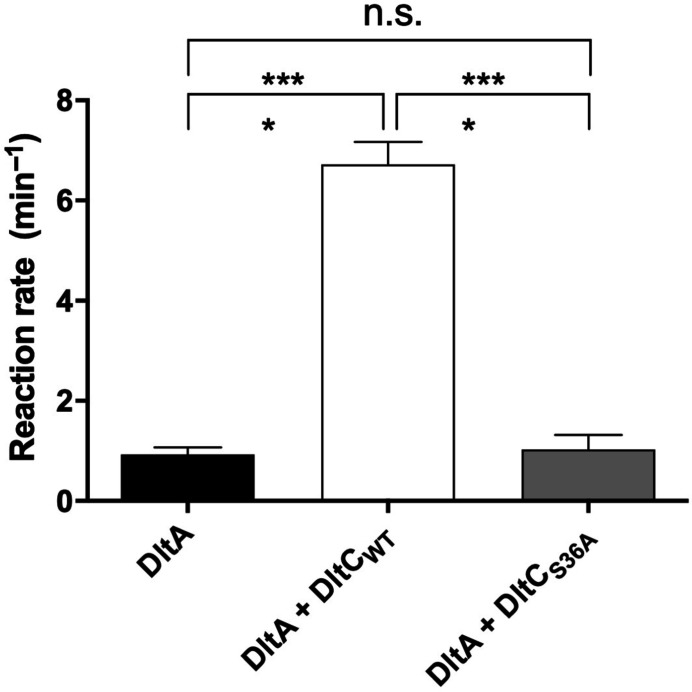
Ppant-modified *Sa*DltC activates *Sa*DltA. Initial rates of adenylation catalyzed by *Sa*DltA (5 µ*M*) in the presence of wild-type *Sa*DltC (300 µ*M*) or the S36A mutant (300 µ*M*). Error bars represent the standard deviation (*n* = 3). Statistical significance was evaluated by one-way ANOVA with Tukey’s multiple comparison test. Statistical values of *p* < 0.05 were considered to be statistically significant. ***, *p* < 0.0001; ns, not significant.

The adenylation activity of *Sa*DltA was increased by approximately 7.2-fold in the presence of wild-type *S. aureus* DltC, but the S36A mutant did not significantly affect the activity (Fig. 6 ▸). These data indicate that *Sa*DltC is able to activate the adenylation activitiy of *Sa*DltA and that the Ppant group covalently attached to DltC is necessary for activation.

## 4. Discussion

Bacterial resistance to antibiotics is a global health emergency that is not only increasing rapidly but is also severe. By 2050, it has been estimated that the total number of deaths caused by antimicrobial resistance will have increased by ten million per year worldwide (O’Neill, 2014 ▸). Among antibiotic-resistant pathogens, *S. aureus* is especially notorious for its unique ability to rapidly become resistant to a broad range of antibiotics, making the pathogen extremely difficult to treat (Chambers & DeLeo, 2009 ▸). Therefore, *S. aureus* resistant to methicillin was classified by the World Health Organization (WHO) as one of 12 ‘priority pathogens’ that pose the greatest threat to human health and for which new antibiotics are urgently needed (Willyard, 2017 ▸).

*S. aureus* has become resistant to antibiotics through numerous strategies, including the inactivation of antibiotics (for example the production of penicillinase, a penicillin-binding protein), cell-wall thickening, spontaneous mutations of topoisomerase IV and DNA gyrase, and overexpression of an efflux pump (for example NorA) (Chambers & DeLeo, 2009 ▸; Lowy, 2003 ▸; Schmitz *et al.*, 1998 ▸; Kaatz & Seo, 1995 ▸; Fishovitz *et al.*, 2014 ▸). In this study, we focused on the d-alanylation process mediated by four proteins (DltA, DltB, DltC and DltD) encoded by the *dlt* operon that have drawn much attention as novel antibiotic drug targets due to their indispensable role in the virulence and survival of *S. aureus* (Weidenmaier *et al.*, 2003 ▸, 2004 ▸; Collins *et al.*, 2002 ▸; Kristian *et al.*, 2003 ▸).

Our crystal structure of *Sa*DltA complexed with ATP and Mg^2+^ reveals detailed interactions involving the P-loop. We observed that the P-loop not only binds to the β- and γ-phosphate moieties of ATP, but also directly binds to residues in the hinge region and Ppant blocking loop in the C-lobe (Figs. 1 ▸ *c* and 1 ▸ *e*). We interpret these observations to indicate that the interactions involving the P-loop may be required for DltA to adopt a properly positioned structure for the adenylation reaction. In the absence of such interactions, as shown in chains *C* and *D*, the overall *B* factor of the C-lobe is significantly higher, supporting our assumption that inter­actions involving the P-loop stabilize the overall adenylation conformation of the protein (Figs. 1 ▸ *d* and 1 ▸ *e*).

In the *Sa*DltA structure, several key interdomain inter­actions (between the N- and C-lobes) that are observed in the adenylation conformation of *B. cereus* DltA are missing (Fig. 3 ▸). This might affect the overall catalytic efficiency of the enzyme in the adenylation reaction, as additional substrate-binding energy is required to compensate for this entropic penalty. Indeed, the catalytic efficiency (*k*_cat_/*K*_m_) of *Sa*DltA for d-alanine is lower than that of *B. cereus* DltA by an order of magnitude (Fig. 5 ▸ *c*; Du *et al.*, 2008 ▸). Interestingly, the cytoplasmic concentration of d-alanine in *S. aureus* reaches approximately 1.5 m*M* in the stationary phase, which is similar to the *K*_m_ value of *Sa*DltA for d-alanine (Fig. 5 ▸ *c*), while *B. subtilis* produces only marginal amounts of d-alanine (Lam *et al.*, 2009 ▸). In addition to this, the enantioselectivity of *Sa*DltA is much higher than that of *B. cereus* DltA (Fig. 5 ▸), suggesting that the rate of the d-alanine-adenylate-forming reaction of *S. aureus in vivo* may be higher than that of *B. cereus*.

The single enzyme DltA catalyzes two sequential reactions: (i) adenylation of d-alanine and (ii) thioesterification of the Ppant moiety attached to DltC. In the presence of Ppant-loaded *Sa*DltC, which is a substrate of the second reaction, the adenylation reaction rate of *Sa*DltA was increased by approximately 7.2-fold, which is in agreement with previous work (Fig. 6 ▸; Wood *et al.*, 2018 ▸). Conceivably, the Ppant-binding pocket in DltA could be exploited for the development of selective inhibitors, although further structural work on Ppant–DltA complexes will be necessary.

Undoubtedly, a better understanding of the enzymes involved in the pathogenicity and virulence of *S. aureus* could be of great help in the design of new antibacterial agents. As DltA is a protein that is essential for the pathogenicity of *S. aureus*, the observed unique structural features as well as the kinetic parameters reported here may be useful in the design of effective antibiotic peptides or small molecules and may accelerate a rational approach for the development of effective antibiotics specific for *S. aureus*.

## Supplementary Material

PDB reference: d-alanine alanyl carrier protein ligase, 7vhv

Supplementary Figures. DOI: 10.1107/S2059798322000547/ji5025sup1.pdf

## Figures and Tables

**Table 1:** Data-collection and refinement statistics The structure was solved with a data set from a single crystal. Values in parentheses are for the highest resolution shell.

Data collection	
Wavelength (Å)	0.9000
Space group	*P*2_1_
*a*, *b*, *c* (Å)	89.47, 88.51, 130.85
α, β, γ (°)	90.0, 91.67, 90.0
Resolution (Å)	50.00–2.55 (2.64–2.55)
*R*_merge_	0.103 (0.527)
〈*I*/σ(*I*)〉	10.3 (7.3)
No. of reflections	333087 (8768)
Completeness (%)	99.5 (98.2)
Multiplicity	5.0 (4.7)
Wilson *B* factor (Å^2^)	40.1
CC_1/2_	0.96 (0.80)
Refinement	
Resolution (Å)	39.91–2.55
No. of unique reflections	66484
Completeness (%)	99.5
*R*_work_/*R*_free_ (%)	18.9/23.9
No. of atoms	
Protein	15270
ATP	124
Mg^2+^	4
Water	381
*B* factors (Å^2^)	
Protein	47.9
ATP	41.9
Mg^2+^	46.7
Water	42.9
R.m.s. deviations	
Bond lengths (Å)	0.012
Bond angles (°)	1.48
Ramachandran plot (%)	
Favored	96.87
Outliers	0.31
PDB code	7vhv
