# Supplementary material for: Structural and functional analysis of the d-alanyl carrier protein ligase DltA from Staphylococcus aureus Mu50
Source: Acta Crystallogr D Struct Biol. 2022 Mar 16;78(Pt 4):424–34. doi: 10.1107/S2059798322000547 (PMC8972799; doi:10.1107/S2059798322000547)
Supplement: Supplementary file 1 [file d-78-00424-sup1.pdf]

STRUCTURAL BIOLOGY

Volume 78 (2022)

Supporting information for article:

Structural and functional analysis of the D-alanyl carrier protein ligase DltA from *Staphylococcus aureus* Mu50

In-Gyun Lee, Chiman Song, Seoyeong Yang, Hanul Jeon, Jingyeong Park, Hye-Jin Yoon, Hookang Im, Sung-Min Kang, Hyun-Jong Eun and Bong-Jin Lee

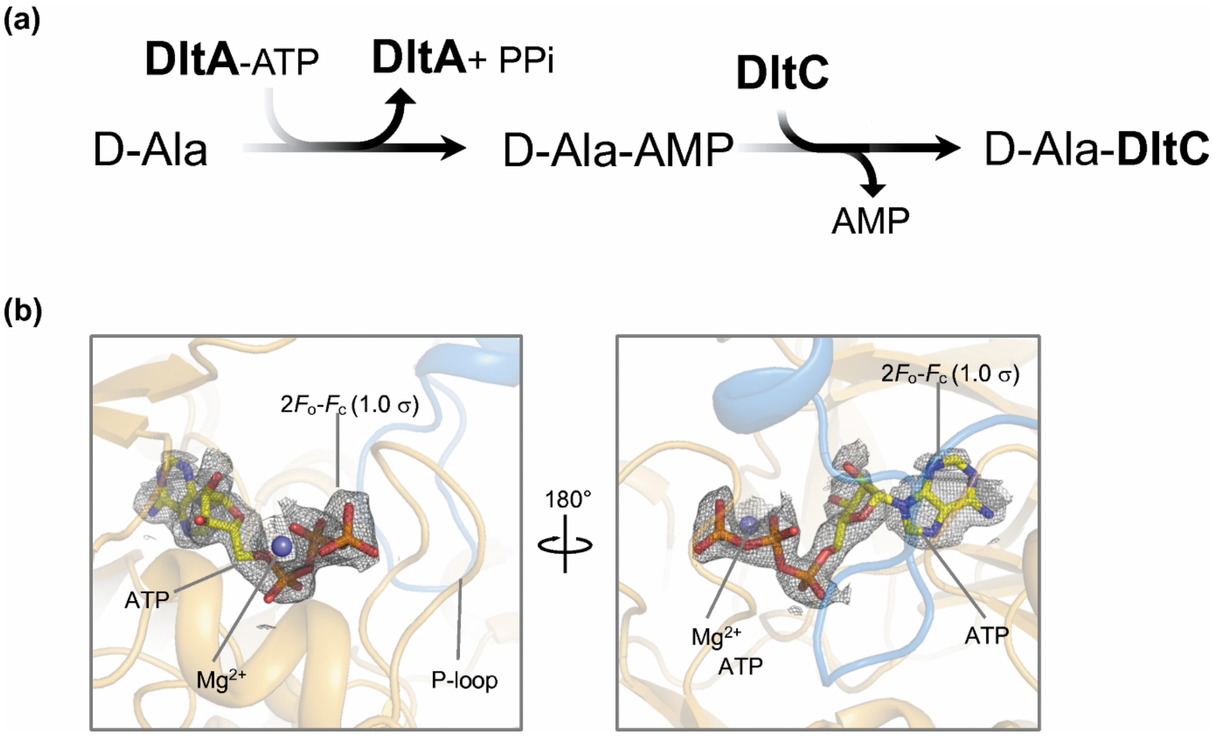

Figure S1 Reaction mechanism catalyzed by DltA and electron density map of ATP and Mg^{2+} molecules bound to *Sa*DltA. (a) Reaction mechanism catalyzed by DltA and DltC. (b) Close-up view showing the ATP and Mg^{2+} bound active site of *Sa*DltA chain A. The *2Fo-Fc* electron density map (gray mesh) contoured at 1.0 σ is shown around ATP and Mg^{2+}.

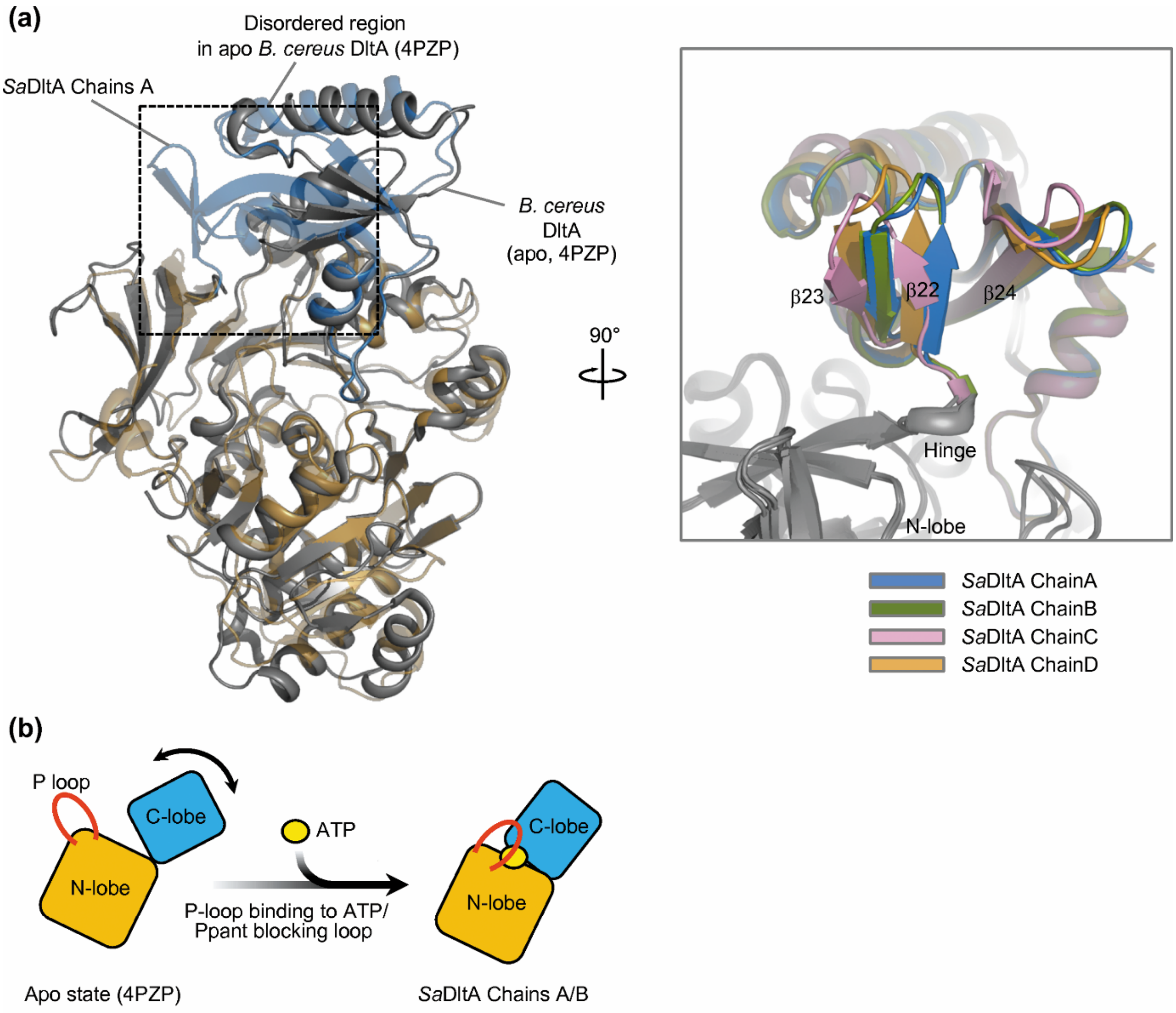

(c)

	Observed residues	ATP molecule	P-loop	Average B factor of C-lobe	r.m.s. deviations (Å)
ChainA	1-485	Observed	Observed	50.2	0.38 (between A and B)
ChainB	1-485	Observed	Observed	45.7	
ChainC	2-147, 152-485	Observed	Not observed	73.6	1.56 (between C and D)
ChainD	2-147, 152-485	Observed	Not observed	64.7	

Figure S2 Comparison of the different copies of the *S. aureus* DltA present in the asymmetric unit. (a) Left: Structural overlay of the *Sa*DltA and *B. cereus* apo DltA (PDB ID: 4PZP). The N- and C-lobes of *Sa*DltA are colored orange and blue, respectively. *B. cereus* apo DltA is colored gray. Dotted box indicates disordered region of C-lobe of the *B. cereus* DltA. Right: Structural overlay of four chains of the *Sa*DlA present in the asymmetric unit. The slightly different orientations and secondary structural elements in the β22, β23 and β24 region suggest that this region is dynamic. (b) Cartoon representation describing the structural transition upon P-loop binding to ATP/Ppant blocking loop. (c) Comparison between chains A–D in the asymmetric unit.

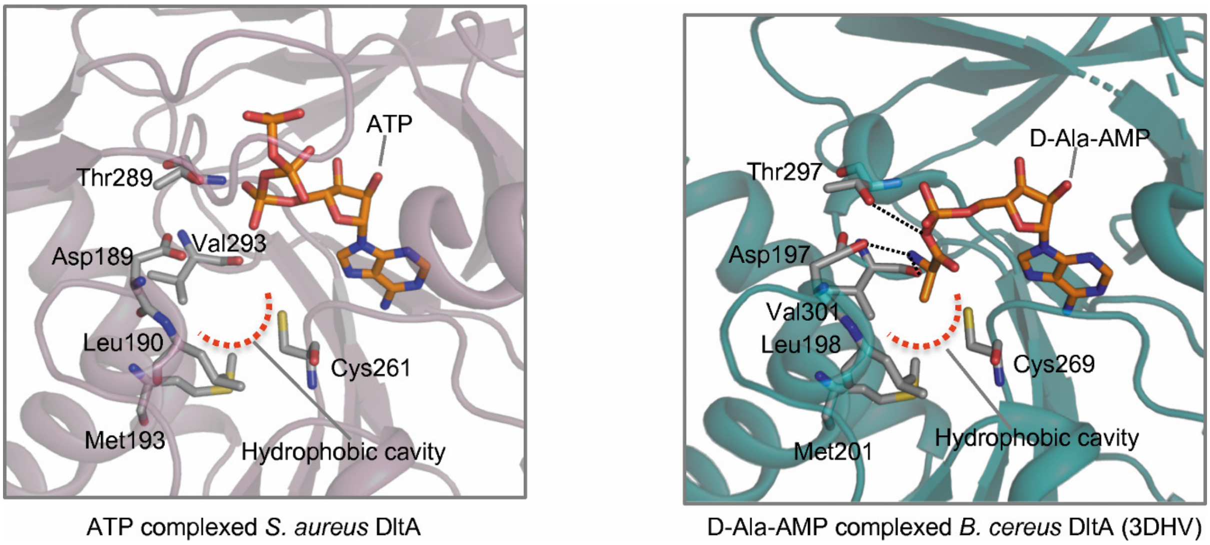

Figure S3 Proposed D-alanine binding site in ATP complexed *Sa*DltA (Left) and comparison with D-Ala-AMP complexed *B. cereus* DltA (Right; PDB ID: 3DHV).

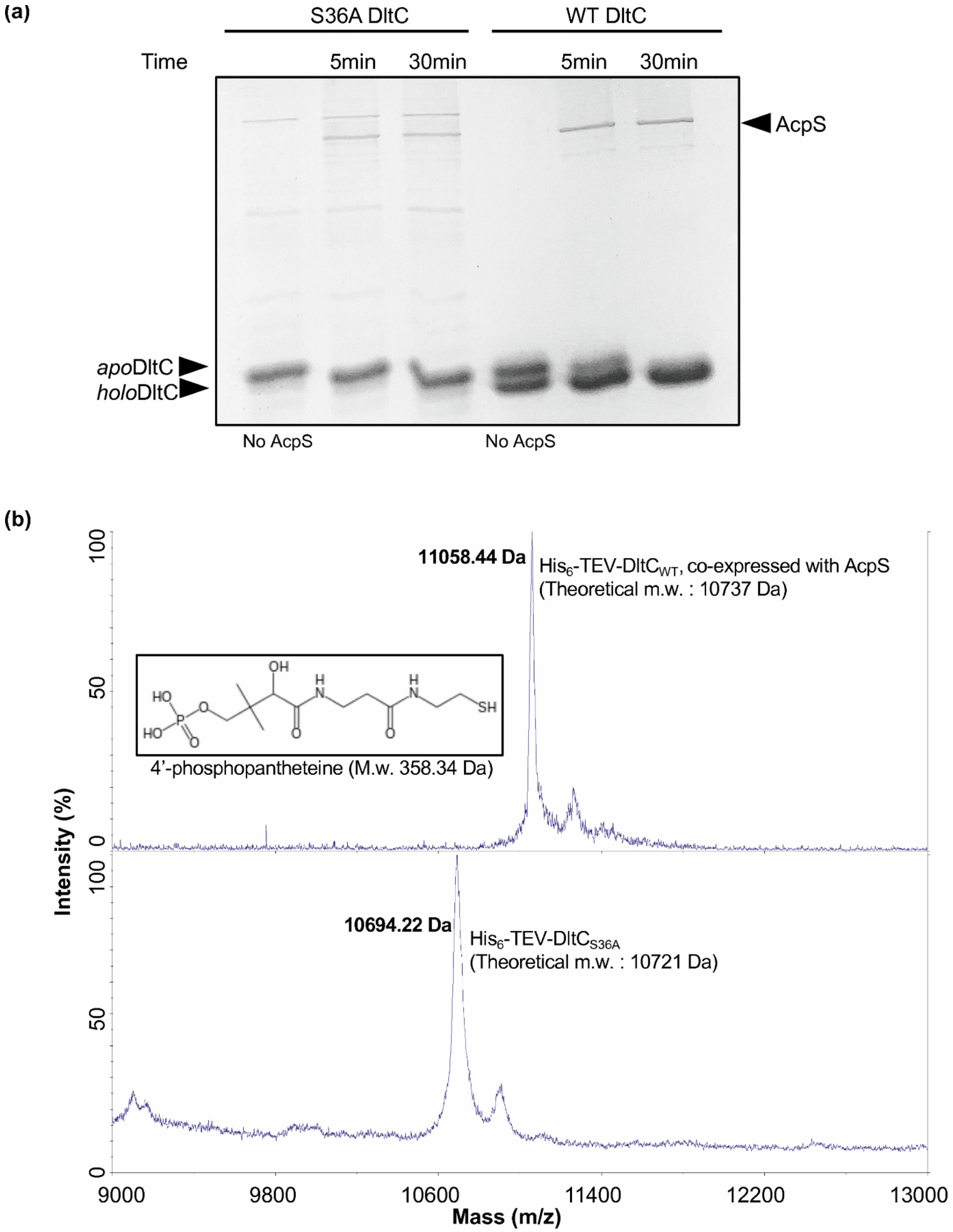

Figure S4 Characterization of Ppant modified WT *Sa*DltC and non-modified S36A *Sa*DltC. (a) Native PAGE analysis of *in vitro* phosphopantetheinylation of purified *Sa*DltC. WT *Sa*DltC purified from *E. coli* migrated as two bands on native PAGE while S36A *Sa*DltC migrated as a single band. Upon AcpS-catalyzed phosphopantetheinylation, WT *Sa*DltC showed a band shift to a single band

(holo DltC). After 5 min and 30 min, reactions containing 100 μM DltC, 1 mM CoA, 1 mM DTT and 25 μM AcpS were electrophoresed and stained with Coomassie blue. (b) MALDI-TOF mass spectra of WT *Sa*DltC and S36A *Sa*DltC. Upper panel: Mass spectrum of the WT *Sa*DltC, co-expressed with AcpS. An increase of 364 Da compared to S36A *Sa*DltC (Lower panel) indicates that 4' ppant-moiety (Inset) is covalently linked to Ser36 of the protein.
